# Glucolipid metabolic dysfunction in ankylosing spondylitis: inflammatory mechanisms, immune–metabolic crosstalk and therapeutic implications of natural products and traditional Chinese medicine

**DOI:** 10.3389/fimmu.2026.1874215

**Published:** 2026-08-10

**Authors:** Xiaofei Dong, Jingjing Zhang, Zixuan Chen, Yeqi Liang, Yi Chen, Zheng Xiao, Yingcun Li, Ke Li

**Affiliations:** 1 Department of Basic Medical Sciences, Gansu University of Chinese Medicine, Lanzhou, Gansu, China; 2 The Second Affiliated Hospital of Guangzhou University of Chinese Medicine, Guangzhou, China; 3 Gansu Provincial Key Research Base for Humanities and Social Sciences, Dunhuang Medical Literature Compilation and Application Research Center, Lanzhou, China; 4 Department of Encephalopathy, Qujing Hospital of Traditional Chinese Medicine, Qujing, China

**Keywords:** ankylosing spondylitis, cytokine, glucolipid metabolism, immune, inflammation

## Abstract

Ankylosing spondylitis (AS) is increasingly recognized as a systemic inflammatory disease accompanied by substantial glucolipid metabolic disturbances and an elevated risk of diabetes. Beyond articular inflammation, persistent immune activation reshapes metabolic homeostasis through interconnected mechanisms involving chronic cytokine signaling, immune-cell metabolic reprogramming, gut microbiota dysbiosis and endocrine disruption. Key mediators, including TNF-α, IL-6 and IL-17, promote insulin resistance, lipid abnormalities and endothelial dysfunction, while Th17/Treg imbalance, neutrophil activation and microbial metabolite alterations further amplify metabolic injury. These pathogenic interactions establish a self-reinforcing cycle in which inflammation and metabolic dysfunction mutually exacerbate one another. Emerging evidence also indicates that anti-inflammatory therapies, nutritional strategies and metabolic modulators may provide dual benefits by controlling disease activity while improving metabolic outcomes. This review summarizes the mechanistic links between AS and glucolipid dysregulation and highlights integrated therapeutic strategies, including pharmacological interventions, nutritional approaches, and natural products/traditional Chinese medicine, for reducing diabetes risk and improving long-term prognosis in AS.

## 1 Introduction

Ankylosing spondylitis (AS), a chronic inflammatory disorder within the spondyloarthritis spectrum, primarily affects the spine and sacroiliac joints (1). Its pathological hallmark is progressive ossification of ligaments, intervertebral discs, endplates and epiphyseal structures (2). AS affects 0.1–1.4% of the global population and occurs approximately twice as often in men as in women (3). Although traditionally defined by musculoskeletal manifestations, AS is increasingly recognized as a systemic disease accompanied by metabolic comorbidities, particularly metabolic syndrome (MetS) (4). Notably, diabetes mellitus has emerged as a clinically important metabolic comorbidity in AS. Epidemiological studies have shown that patients with AS have an increased risk of Type 2 Diabetes Mellitus (T2DM), with incidence rates reported to be 1.17-fold higher than those in non-AS populations (5). More recent evidence further supports this association: a population-based study reported a higher incidence of diabetes among patients with AS than among individuals without AS, while a systematic review and meta-analysis of observational studies found that diabetes was more prevalent in axial spondyloarthritis and that the overall odds of diabetes were significantly increased compared with non-axial spondyloarthritis populations (6). In addition, Mendelian randomization analyses have provided emerging genetic evidence suggesting a potential causal relationship between AS and diabetes-related traits, including altered blood glucose, fasting glucose and insulin levels (7). Conversely, diabetes itself may increase susceptibility to AS, as indicated by genetic evidence linking type 1 diabetes mellitus to a higher risk of AS (8). Mechanistically, this bidirectional association is thought to arise from sustained low-grade inflammation, immune overactivation, disrupted insulin signaling, abnormal adipokine secretion, gut microbiota dysbiosis and endocrine disturbance, underscoring the need for strengthened glucose monitoring, early diabetes screening and integrated metabolic management in AS (9). In AS, these abnormalities commonly manifest as elevated triglyceride and LDL levels, which are closely intertwined with chronic inflammation (10, 11). Through a highly interconnected immune network, AS-associated inflammatory cells and mediators disrupt glucose and lipid metabolism in the liver, adipose tissue, skeletal muscle and pancreas, thereby promoting insulin resistance and systemic metabolic dysfunction (12). In turn, these metabolic abnormalities further amplify inflammatory responses (13), establishing a self-perpetuating pathogenic cycle. This review summarizes the mechanisms linking AS to glucolipid metabolic dysfunction, with particular emphasis on chronic inflammation, immune dysregulation, endocrine disturbance and impairment of the gut microbial–metabolic axis. We further propose an integrated immune–metabolic framework in which AS-related inflammation, immune-cell imbalance, gut microbiota dysbiosis and metabolic dysfunction interact to form a self-reinforcing cycle that may increase diabetes risk and worsen long-term cardiometabolic outcomes.

This review was prepared as a narrative synthesis. Relevant studies were identified through PubMed, Web of Science and Google Scholar using terms related to ankylosing spondylitis, axial spondyloarthritis, diabetes, insulin resistance, glucose and lipid metabolism, inflammation, immune regulation and gut microbiota. Priority was given to human studies, meta-analyses, Mendelian randomization studies and AS-related translational research. Evidence from diabetes, obesity, other inflammatory diseases or experimental models was included only when AS-specific data were limited and was interpreted as supportive mechanistic evidence.

## 2 Abnormal glucolipid metabolism in patients with ankylosing spondylitis

Inflammation-driven abnormalities in glucose and lipid metabolism substantially increase cardiovascular risk in patients with ankylosing spondylitis (AS). In recent years, epidemiological evidence addressing metabolic disturbances in AS has expanded considerably (14–16). The association between AS and cardiovascular disease is particularly striking (17). Cardiovascular morbidity is consistently higher in patients with AS than in the general population, a pattern likely driven by both inflammation-accelerated atherosclerosis and direct cardiac involvement, including aortitis and conduction abnormalities (18). This risk appears especially pronounced in patients with earlier disease onset and heightened inflammatory burden, as indicated by increased C-reactive protein (CRP) levels (19). Beyond cardiovascular complications, the relationship between AS and diabetes is increasingly recognized as bidirectional, with chronic inflammation serving as a central mechanistic hub (6). Moreover, large-scale Mendelian randomization analyses have provided evidence for a causal relationship between AS and diabetes, further linking AS to alterations in blood glucose, fasting glucose and insulin levels (7). As disease activity increases, patients with AS exhibit marked elevation of pro-inflammatory cytokines, particularly TNF-α, IL-17 and IL-6, which not only aggravate joint inflammation but also directly or indirectly drive the initiation and progression of diabetes through multiple pathogenic pathways (20). Collectively, these findings support the view that persistently elevated inflammatory mediators constitute a key bridge between AS and diabetes. Accordingly, effective suppression of systemic inflammation should be regarded as a central component of metabolic management in AS, ideally combined with personalized therapeutic strategies to improve long-term outcomes. Notably, diabetes itself is also associated with an increased risk of AS, a link that may be mediated by chronic systemic inflammation, genetic predisposition and environmental influences (8). These observations further underscore the importance of timely glycemic control in the comprehensive management of AS.

## 3 AS-induced dysregulation of glucose and lipid metabolism

### 3.1 The central role of chronic inflammation

Chronic inflammation is a central driver of diabetes pathogenesis. Accumulating evidence indicates that elevations in inflammatory biomarkers are already detectable during the earliest stages of diabetes, even before overt clinical manifestation or definitive diagnosis (21). These observations support the concept that low-grade chronic inflammation is not merely a consequence of metabolic dysfunction, but may instead function as an initiating event in diabetes development. In ankylosing spondylitis (AS), persistent immune activation further disrupts the inflammatory cytokine network, as reflected by markedly increased circulating levels of key mediators such as TNF-α and IL-6 (22). Through multiple interconnected pathways, these inflammatory signals profoundly perturb glucose and lipid homeostasis.

**Figure 1:**
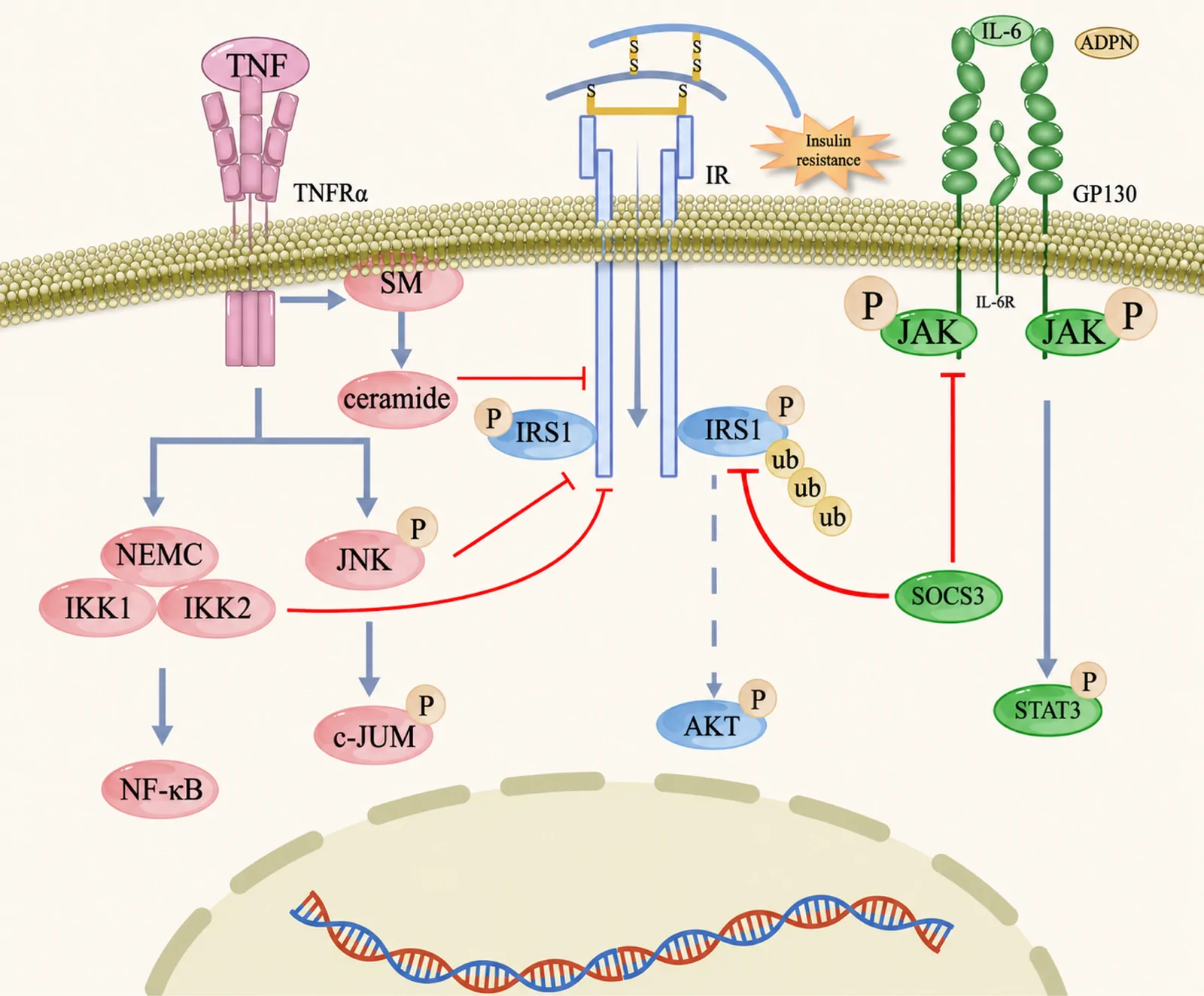
Inflammatory signaling and glucolipid metabolism in insulin resistance. TNF-α plays a pivotal role in the development of insulin resistance through multiple signaling pathways. As depicted in the diagram, TNF-α stimulates the NF-κB pathway via IKKβ and activates the JNK pathway. This activation interferes with the tyrosine phosphorylation of IRS-1, a critical step in insulin signaling, thereby inducing insulin resistance. IL-6 exerts its effects through the STAT3 pathway, leading to the downregulation of SLC39A5. This downregulation results in a decreased concentration of zinc, which in turn affects the ATP channel and enhances glucagon release, further contributing to metabolic dysregulation. TNF-α suppresses the expression of adiponectin, an adipokine with anti-inflammatory and insulin-sensitizing properties. Concurrently, it increases the production of pro-inflammatory factors, leading to an imbalance in adipokine levels. This imbalance, coupled with the aforementioned signaling disruptions, culminates in insulin resistance, a hallmark of metabolic disorders such as T2DM.

Among these mediators, TNF-α occupies a pivotal position in AS-associated inflammation. As a master pro-inflammatory cytokine, TNF-α activates IKK-β within the IκB kinase complex, thereby initiating the nuclear factor-κB (NF-κB) signaling cascade. In parallel, TNF-α also stimulates the c-Jun N-terminal kinase (JNK) pathway. Coordinated activation of these two canonical inflammatory pathways amplifies the production of downstream inflammatory mediators, generating a self-sustaining circuit that perpetuates chronic inflammation. Importantly, this inflammatory cascade interferes with the tyrosine phosphorylation of insulin receptor substrate-1 (IRS-1), thereby impairing its ability to transmit downstream insulin signals. The resulting signaling defect markedly weakens insulin-mediated metabolic regulation, ultimately leading to insulin resistance, reduced glucose uptake and impaired glucose metabolism, thereby increasing susceptibility to type 2 diabetes and related metabolic disorders (23). IL-6 also exerts substantial metabolic effects. Notably, IL-6 shows a positive association with glucagon rather than insulin. Mechanistically, IL-6 activates signal transducer and activator of transcription 3 (STAT3), which suppresses expression of the zinc transporter SLC39A5, a key regulator of intracellular zinc homeostasis. Reduced cytoplasmic free zinc availability aggravates ATP channel dysfunction, diminishes channel activity, enhances membrane depolarization and promotes calcium influx, thereby stimulating glucagon secretion and increasing diabetes risk (24). These findings highlight that inflammatory cytokines can influence glucose metabolism not only through insulin resistance, but also through direct modulation of endocrine pathways governing glucagon release (**Figure 1**).

Inflammation also disrupts adipokine homeostasis, thereby linking immune activation to lipid metabolic dysfunction. Pro-inflammatory mediators such as TNF-α suppress adiponectin expression and secretion while simultaneously promoting the production of additional inflammatory cytokines through multiple signaling pathways (25). This shift in the cytokine milieu reflects the dysfunctional state of hypertrophic adipocytes within an inflammatory microenvironment. Adiponectin, an adipocyte-derived hormone with pleiotropic biological activities, exerts insulin-sensitizing, anti-diabetic, anti-obesity, anti-inflammatory, anti-atherosclerotic, cardioprotective and neuroprotective effects (26). Therefore, inflammation is not simply an isolated immune event; rather, it amplifies its own metabolic consequences by reshaping adipokine expression and secretion, thereby aggravating lipid dysregulation and insulin resistance. At the tissue level, this process is further reinforced by inflammatory remodeling of adipose tissue. The accumulation of pro-inflammatory macrophages in adipose depots impairs adipose function by suppressing adipogenesis, intensifying local inflammation, inducing insulin resistance and promoting fibrotic remodeling (27). This establishes a pathological cycle in which inflammation and metabolic imbalance are mutually reinforcing. In parallel, inflammatory signals can directly stimulate lipolysis. Studies have shown that lipopolysaccharide (LPS) induces hormone-sensitive lipase (HSL) phosphorylation through both the MEK/ERK cascade and the canonical protein kinase A (PKA) pathway, thereby enhancing enzymatic activity and accelerating triglyceride hydrolysis in adipocytes. This process results in the rapid release of large quantities of free fatty acids (FFA) into the circulation, accompanied by increased expression of pro-inflammatory cytokines such as IL-6 and IL-8. At the same time, insulin sensitivity in adipose tissue is impaired through suppression of Akt phosphorylation (28).

### 3.2 Immune cells and metabolic crosstalk

Immune dysregulation in AS extends beyond perturbations in inflammatory cytokine networks and encompasses profound metabolic reprogramming across immune cell subsets and their interactions with metabolic tissues. Among these alterations, disruption of the Th17/Treg axis is a central pathogenic feature of ankylosing spondylitis (29). Th17 cells sustain their pro-inflammatory identity through aerobic glycolysis, which supports both clonal expansion and IL-17 production at inflamed sites such as the joints. Glycolytic by-products, particularly lactate, further intensify local inflammation and may contribute to broader systemic metabolic disturbance under chronic inflammatory conditions (30). By contrast, Treg differentiation and suppressive activity rely predominantly on oxidative phosphorylation and mitochondrial reactive oxygen species signaling, which are essential for stabilizing Foxp3 expression and maintaining anti-inflammatory cytokine production (31). Thus, the Th17/Treg imbalance in AS is characterized by expansion of Th17 cells and their associated mediators, including IL-17, IL-6, IL-23 and RORγt, together with reduction of Treg cells and related factors such as IL-10, TGF-β and Foxp3 (32).

Mechanistically, persistent exposure to pro-inflammatory cytokines, particularly IL-6 and TNF-α, suppresses FOXP3 expression or induces epigenetic instability, including aberrant methylation within the FOXP3 promoter TSDR, thereby weakening Treg suppressive capacity (33). Under these conditions, a subset of Treg cells may acquire a Th17-like phenotype marked by RORC expression and IL-17 production, creating a self-reinforcing loop of inflammation and immune dysfunction that accelerates joint damage (33). Gut microbiota-derived metabolites further shape this axis. In particular, tryptophan metabolites produced by certain eukaryotic microorganisms can promote Th17 differentiation while constraining Treg development and function, thereby undermining immune tolerance and sustaining chronic inflammation in autoimmune disorders such as AS (34). Collectively, these findings identify the Th17/Treg disequilibrium as a metabolically and immunologically coordinated driver of AS pathogenesis. Neutrophils have also emerged as important contributors to AS. Neutrophil-derived exosomes, particularly TNF-Exo, activate NF-κB signaling and enhance NLRP3 inflammasome activity, thereby amplifying inflammatory responses (35). Notably, neutrophils display stage-dependent functional duality. Early in disease, they can traverse the intestinal epithelial barrier to limit mucosal inflammation and facilitate repair, whereas sustained activation at later stages disrupts barrier integrity through excessive infiltration, apoptosis and continued release of pro-inflammatory mediators, ultimately promoting tissue injury and delaying recovery (36). Consistent with this pathogenic role, AS-associated neutrophils express high levels of macrophage migration inhibitory factor and HIF1A, both of which are tightly linked to inflammatory activation (37, 38). Moreover, neutrophil-derived proteases such as elastase can directly impair insulin sensitivity, thereby aggravating disordered glucose metabolism (39) (**Table 1**).

**Table 1:** The pathological mechanisms of AS-related glucolipid metabolic disorders.

Mechanisms of pathology	Key effector molecules	Metabolic effects	Potential for therapeutic targets
Sustained production of chronic inflammatory mediators	TNF-α, IL-6	Insulin signaling is suppressed, adipose tissue dysfunction, and glucagon secretion is increased	TNF Inhibitors
Th17/Treg imbalance	Th17, indole	Th17 aggravated joint injury and impaired Treg function	IL-23 antagonist, microbiome modulation
Pathogenic neutrophils	HIF1A, MIF	Promotes osteogenic differentiation and reduces insulin sensitivity	HIF1A inhibitor and MIF antagonist
Dysbiosis of intestinal flora	Short chain fatty acids, bile acids	Intestinal barrier disruption and bile acid metabolism disorder	Probiotics, prebiotics, microbiota transplantation
Adipokine imbalance	Adiponectin	Decreased insulin sensitivity, chronic inflammatory state	Adiponectin analogues, leptin sensitizers

### 3.3 Disruption of the gut microbiota–metabolism axis

Gut microbiota dysbiosis, a major environmental determinant of AS, is increasingly recognized as a crucial link between immune activation and metabolic dysfunction. In patients with AS, persistent inflammasome activation appears to be mechanistically associated with microbial imbalance (40). This dysbiosis is characterized by reduced microbial diversity, depletion of beneficial taxa with immunoregulatory and barrier-protective functions, such as *Bacteroides*, and expansion of opportunistic pathogens, including *Proteobacteria* and *Streptococcus* (41). Such ecological disruption compromises intestinal homeostasis, increases gut permeability and promotes the release of inflammatory mediators, thereby facilitating AS progression. Moreover, reduced production of short-chain fatty acids weakens epithelial barrier integrity, disturbs immune equilibrium—particularly the Th17/Treg axis—and diminishes anti-inflammatory capacity (42). Microbiota-dependent metabolic disruption in AS also involves bile acid and tryptophan pathways. Altered microbial composition perturbs bile acid homeostasis and attenuates signaling through Farnesoid X Receptor and G protein-coupled bile acid receptor 1, thereby impairing the regulation of glucose and lipid metabolism (43, 44). By contrast, indole, a tryptophan-derived microbial metabolite, exerts immunoregulatory effects by activating aryl hydrocarbon receptor signaling, promoting FoxP3 expression while suppressing STAT3 and RORγt, thus helping to restore Th17/Treg balance and restrain inflammation (45). However, dysbiosis also suppresses microbial genes involved in carbohydrate biosynthesis and metabolism, contributing to broader metabolic disequilibrium (46). In parallel, microbial products such as lipopolysaccharide may disrupt intestinal barrier integrity and amplify systemic inflammatory signaling, thereby providing an additional mechanistic route linking AS to diabetes and related metabolic disorders.

## 4 Clinical management and treatment strategies

Given the chronic and progressive nature of ankylosing spondylitis, in conjunction with the extensively characterized association between systemic inflammation and metabolic disorders, standardized metabolic monitoring should be systematically incorporated into disease management protocols. Current guidelines recommend that all AS patients undergo annual comprehensive metabolic assessments, including evaluation of glycemic control, lipid profiles, blood pressure, and BMI. For high-risk subgroups-particularly those with pre-existing metabolic conditions (such as diabetes or dyslipidemia) or receiving glucocorticoid therapy-more intensive monitoring at 6-month intervals is advised due to their significantly elevated metabolic risk profile. This structured follow-up approach not only enables early detection of metabolic deterioration but also facilitates timely therapeutic interventions and preventive strategies.

### 4.1 Ameliorative effect of anti-inflammatory treatment on metabolism

Effective suppression of inflammatory activity in AS may also confer metabolic benefit. Among currently available therapies, the IL-17 axis has emerged as a particularly important target, given its central role in autoimmune and inflammatory pathogenesis. Accordingly, multiple IL-17 inhibitors have been developed and approved for immune-mediated diseases, including psoriasis, psoriatic arthritis and AS (47). These agents have shown sustained efficacy in reducing disease activity, even in patients with inadequate responses to TNF inhibitors, while generally maintaining an acceptable safety profile (48). TNF-α inhibitors are likewise well established in the treatment of AS and other chronic inflammatory disorders, including rheumatoid arthritis and Crohn’s disease (49). Beyond controlling inflammation, these agents may improve insulin resistance by restoring insulin signaling and modulating metabolic regulators such as adiponectin and IRS proteins (50). Such findings suggest that TNF blockade may provide dual benefit by alleviating articular inflammation while partially correcting metabolic dysfunction. Nevertheless, TNF inhibitor exposure has also been associated with a 36% increased risk of inflammatory central nervous system disorders, irrespective of disease type or specific agent, which warrants clinical caution (51). JAK inhibitors offer an additional targeted strategy through modulation of JAK–STAT signaling and have expanded the therapeutic landscape for autoimmune disease (52). Notably, tofacitinib appears to influence lipid metabolism by promoting cholesterol efflux to HDL and apolipoprotein A-I, reducing cholesterol uptake from serum, and limiting intracellular cholesterol accumulation (53). These observations indicate that anti-inflammatory therapies in AS may exert broader metabolic effects, although their long-term risk–benefit profiles require careful assessment.

### 4.2 Nutritional intervention reduces inflammation in ankylosing spondylitis

Nutritional intervention should take into account both anti-inflammatory and metabolic benefits. The Mediterranean diet, defined by the regular consumption of extra-virgin olive oil, legumes, whole grains, nuts, fruits, vegetables, fish, dairy products, and moderate wine intake, is advocated as a beneficial nutritional strategy for patients with AS. For patients with AS, this dietary pattern not only contributes to the alleviation of systemic inflammation but also supports weight control and improves metabolic parameters, thereby reducing cardiovascular risk and enhancing overall quality of life. However, AS patients require particular dietary vigilance against high-sugar beverages and ultra-processed foods. These products typically contain refined sugars, trans fats, excessive sodium, and food additives-characteristics associated with low nutrient density that may trigger glycemic fluctuations, lipid metabolism disorders, and systemic inflammation exacerbation through pro-inflammatory pathway activation (54). Therefore, to better control inflammation, improve metabolic status, and reduce complication risks, AS patients should limit consumption of these unhealthy foods while adopting anti-inflammatory, nutritionally balanced dietary patterns such as the Mediterranean diet.

### 4.3 Hypoglycemic drugs and metabolic modulators

When AS is accompanied by clinically confirmed glucose or lipid metabolic abnormalities, hypoglycemic agents and metabolic modulators may provide additional benefit as part of standard metabolic care; however, these interventions should be considered supportive strategies for comorbidity management rather than AS-specific disease-modifying treatments. Metformin improves insulin sensitivity in peripheral tissues and suppresses hepatic gluconeogenesis, thereby ameliorating insulin resistance. It also favorably modulates lipid metabolism by reducing serum triglycerides and LDL-C, and confers vascular protection under metabolically dysregulated conditions through attenuation of advanced glycation end-product formation, ROS generation, oxidative stress and endothelial dysfunction (55). GLP-1 receptor agonists represent another important therapeutic class. Through glucose-dependent stimulation of insulin secretion and suppression of glucagon release, these agents improve both fasting and postprandial glycemia. They also delay gastric emptying, enhance satiety and reduce food intake, thereby supporting weight reduction (56). SGLT2 inhibitors reduce blood glucose by blocking sodium–glucose co-transporter 2-mediated glucose reabsorption in the proximal renal tubule, thereby promoting glycosuria. Beyond glycemic control, they provide benefits in weight reduction, blood pressure regulation and cardiovascular protection (57). Lipid-lowering therapy should be tailored to the patient’s metabolic profile, with statins remaining a key option for patients with elevated LDL-C (58). Collectively, these pharmacological strategies highlight the importance of integrated metabolic management in AS patients with coexisting glucose and lipid abnormalities.

### 4.4 Natural products and traditional Chinese medicine targeting glucolipid metabolism in AS

Natural products, including bioactive compounds and traditional herbal formulations, have attracted increasing attention as complementary therapeutic approaches for chronic inflammatory diseases due to their multi-target regulatory properties. Although direct clinical evidence demonstrating that natural products improve glucolipid metabolic abnormalities specifically in AS remains insufficient, accumulating experimental evidence suggests that several natural compounds may simultaneously regulate inflammatory responses and metabolic homeostasis, providing potential strategies for targeting inflammation-associated metabolic dysfunction.

Curcumin, a major polyphenolic compound derived from *Curcuma longa*, is one of the most extensively investigated natural compounds with both anti-inflammatory and metabolic regulatory properties. Curcumin suppresses NF-κB activation and reduces the production of pro-inflammatory cytokines, including TNF-α and IL-17. Meanwhile, curcumin improves metabolic disorders through activation of AMP-activated protein kinase (AMPK), enhancement of insulin sensitivity, inhibition of lipid accumulation and regulation of adipokine secretion. These properties suggest that curcumin may represent a potential candidate for modulating the inflammatory metabolic interaction involved in AS progression (59, 60). Resveratrol, another widely studied natural polyphenol, exhibits metabolic protective effects through activation of the SIRT1/AMPK signaling pathway, improvement of mitochondrial function and regulation of glucose and lipid metabolism. Previous studies have demonstrated that resveratrol supplementation can improve insulin sensitivity and lipid profiles, suggesting its potential value in inflammatory diseases accompanied by metabolic abnormalities (61).

Tripterygium wilfordii-derived compounds, particularly triptolide, have demonstrated significant immunomodulatory effects in autoimmune and inflammatory diseases. Triptolide suppresses inflammatory cytokine production through regulation of NF-κB and other immune-related signaling pathways. In AS-related studies, Tripterygium wilfordii preparations have been reported to reduce disease activity and inflammatory biomarkers, indicating their potential anti-inflammatory benefits. However, whether these compounds directly regulate glucolipid metabolism in AS requires further investigation (62).

Traditional herbal formulations have also been explored as complementary approaches for AS management. Duhuo Jisheng Decoction, a classical prescription widely used for rheumatic disorders, has demonstrated anti-inflammatory and immunomodulatory effects through regulation of inflammatory mediators and immune responses. Furthermore, emerging evidence suggests that traditional herbal medicines may influence systemic metabolism through modulation of oxidative stress and the gut microbiota–immune axis, although their specific metabolic effects in AS remain to be clarified (63, 64).

Nevertheless, compared with biologic therapies and metabolic drugs, current evidence regarding natural products in AS remains mainly derived from experimental studies. Further well-designed clinical trials are required to determine their efficacy, safety and potential role in integrated immune–metabolic management of AS.

## 5 Conclusion

Ankylosing spondylitis should no longer be viewed solely as a disease of the axial skeleton, but rather as a systemic immune-inflammatory disorder with substantial metabolic consequences. Accumulating evidence indicates that chronic inflammation, immune-cell metabolic reprogramming, gut microbiota dysbiosis and endocrine imbalance converge to disrupt glucose and lipid homeostasis, thereby increasing susceptibility to insulin resistance, diabetes, dyslipidemia and cardiovascular complications. In this context, inflammatory cytokines such as TNF-α, IL-6 and IL-17 act not only as drivers of joint pathology but also as central mediators linking immune dysregulation to metabolic deterioration.

A deeper understanding of these shared mechanisms has important clinical implications. Routine metabolic surveillance should be incorporated into long-term AS management, particularly in patients with high inflammatory burden or pre-existing metabolic risk factors. Moreover, treatment strategies should extend beyond control of musculoskeletal symptoms to include metabolic protection. Anti-inflammatory biologics, dietary intervention and glucose- or lipid-lowering therapies may together provide a more comprehensive therapeutic framework. Given the emerging nature of this field, future studies should adopt more rigorous and evidence-based designs, including large-scale prospective cohorts, longitudinal metabolic profiling, mechanistic experimental models and well-designed interventional trials. Such studies are needed to clarify the causal pathways linking AS with diabetes and glucolipid metabolic dysfunction, identify reliable biomarkers for early risk stratification, and develop personalized therapeutic strategies that simultaneously target inflammation and metabolic abnormalities. These efforts will be essential for improving systemic health and long-term outcomes in patients with AS.
